# Canagliflozin protects against sepsis capillary leak syndrome by activating endothelial α1AMPK

**DOI:** 10.1038/s41598-021-93156-1

**Published:** 2021-07-01

**Authors:** Marine Angé, Julien De Poortere, Audrey Ginion, Sylvain Battault, Mélanie Dechamps, Giulio G. Muccioli, Martin Roumain, Johann Morelle, Sébastien Druart, Thomas Mathivet, Luc Bertrand, Diego Castanares-Zapatero, Sandrine Horman, Christophe Beauloye

**Affiliations:** 1 Pôle de Recherche Cardiovasculaire (CARD), Institut de Recherche Expérimentale et Clinique (IREC), Université catholique de Louvain (UCLouvain), 55, Avenue Hippocrate B1.55.03.1322, 1200 Brussels, Belgium; 2 Division of Pediatrics, Cliniques Universitaires Saint Luc, 1200 Brussels, Belgium; 3 Division of Intensive Care, Cliniques Universitaires Saint Luc, 1200 Brussels, Belgium; 4 Bioanalysis and Pharmacology of Bioactive Lipids Research Group, Louvain Drug Research Institute (LDRI), Université catholique de Louvain (UCLouvain), 1200 Brussels, Belgium; 5 Division of Nephrology, Cliniques Universitaires Saint-Luc, 1200, Brussels, Belgium; 6 Pôle Nephrologie (NEFR), Institut de Recherche Expérimentale et Clinique (IREC), Université catholique de Louvain (UCLouvain), 1200 Brussels, Belgium; 7 Inserm U970, Paris Cardiovacular Research Center, 75015 Paris, France; 8 Division of Cardiology, Cliniques Universitaires Saint-Luc, 1200, Brussels, Belgium

**Keywords:** Phosphorylation, Bacterial infection, Experimental models of disease, Preclinical research, Translational research

## Abstract

Sepsis capillary leak syndrome (SCLS) is an independent prognostic factor for poor sepsis outcome. We previously demonstrated that α1AMP-activated protein kinase (α1AMPK) prevents sepsis-induced vascular hyperpermeability by mechanisms involving VE-cadherin (VE-Cad) stabilization and activation of p38 mitogen activated protein kinase/heat shock protein of 27 kDa (p38MAPK/HSP27) pathway. Canagliflozin, a sodium-glucose co-transporter 2 inhibitor, has recently been proven to activate AMPK in endothelial cells. Therefore, we hypothesized that canagliflozin could be of therapeutic potential in patients suffering from SCLS. We herein report that canagliflozin, used at clinically relevant concentrations, counteracts lipopolysaccharide-induced vascular hyperpermeability and albumin leakage in wild-type, but not in endothelial-specific α1AMPK-knockout mice. In vitro, canagliflozin was demonstrated to activate α1AMPK/p38MAPK/HSP27 pathway and to preserve VE-Cad’s integrity in human endothelial cells exposed to human septic plasma. In conclusion, our data demonstrate that canagliflozin protects against SCLS via an α1AMPK-dependent pathway, and lead us to consider novel therapeutic perspectives for this drug in SCLS.

Sepsis is a major health concern worldwide^1^, and is defined as a syndrome of dysregulated host response to infection causing life-threatening organ dysfunction^2^. Despite significant advances in the understanding of the disease, the therapeutic management of septic patients primarily relies on supportive care and mortality rates remain unacceptably high, around 40%^3^. Sepsis capillary leak syndrome (SCLS), mainly caused by vascular hyperpermeability, is a critical process in sepsis pathophysiology and has been demonstrated to be an independent prognostic factor of survival^4^. Moreover, growing evidence supports that maintenance of vascular barrier integrity improves sepsis outcome^5–11^. However, no therapeutic proposal that targets SCLS has so far reached the clinical trial stage.

SCLS is caused by vascular barrier disruption. Under healthy conditions, endothelial cells are sealed to one another by inter-endothelial junctions (IEJs) that effectively control the passage of molecules in a size-selective manner. Vascular endothelial cadherin (VE-Cad), the major component of adherens junctions (AJs), is a protein essentially involved in this regulation^12,13^. Its stability depends on the actin cytoskeleton^14^, whose polymerization is notably regulated by the phosphorylation of heat-shock protein of 27 kDa (HSP27), downstream of the p38 MAP kinase (p38MAPK)^15^. Upon sepsis, stress mediators trigger signaling cascades that induce actin cytoskeleton contraction, AJs disruption, and loss of endothelial barrier function^16–18^. This event is characterized by the formation of intercellular gaps, leading to plasma leaking through the endothelium and resulting in widespread edema^18^. Albumin, the main determinant of plasmatic oncotic pressure^19^, notably drives this process through osmotic forces. In addition to dramatically reducing circulating blood volume, capillary leaking directly compromises the microcirculation^20^. First, the fluid accumulating in the interstitial space mechanically compresses capillaries and, thus, impairs microvascular blood flow. Second, the perivascular fluid enhances the distance required for oxygen diffusion. Impaired tissue perfusion and oxygenation processes progressively induce organ failure and ultimately affect patient survival^21,22^.

The catalytic subunit of AMP-activated protein kinase (AMPK) is primarily expressed under its α1-isoform within the microvascular endothelium; there, it acts as a major regulator of the actin cytoskeleton and IEJs^23–25^. Our team and others have previously demonstrated the pivotal role of α1AMPK in the maintenance of endothelial barrier function, in models of endotoxemia^5,26,27^. In mechanistic terms, we demonstrated that endothelial barrier protection by α1AMPK was mediated by p38MAPK/HSP27-dependent enhancement of VE-Cad stability^25^. Nevertheless, none of the AMPK activators used in the different studies can be safely employed in vivo or securely administered to septic patients^5,8,27,28^. In this context, a particularly interesting therapeutic strategy would be to identify AMPK activators that are clinically usable and protect against sepsis-induced vascular leakage.

Canagliflozin, an inhibitor of sodium-glucose co-transporter 2 (SGLT2i), is currently prescribed as oral glucose-lowering agent to patients with diabetes. Independently of modulating glucose transport, clinically relevant canagliflozin concentrations also activate AMPK in different cell types, including human endothelial cells^29^. Interestingly, in addition to increasing renal glucose excretion, strong evidence supports that canagliflozin exerts significant cardiovascular protective effects, whose exact mechanisms are still poorly understood^30–32^. On account of its effect on AMPK activity, we hypothesized that canagliflozin may constitute a new therapeutic option to target SCLS.

In the current study, we have evaluated in vivo the potential benefits of canagliflozin-induced AMPK activation on LPS-induced capillary leak. Using a murine model of specific and conditional endothelial α1AMPK deletion, we have demonstrated that canagliflozin protects against vascular leakage by mechanisms that are dependent upon endothelial α1AMPK. This protection involves both activation of p38MAPK/HSP27 pathway and preservation of VE-Cad integrity. By validating these results in endothelial cells submitted to human plasma collected from septic shock patients, we have laid the groundwork for further clinical investigations.

## Results

### Generation and validation of conditional e-AMPK KO mice

**Figure 1:**
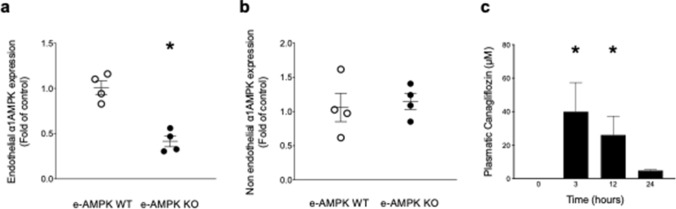
Generation and validation of the experimental model. e-AMPK WT/KO mice were intraperitoneally administered Tamoxifen (500 µg/mice) for 5 consecutive days in order to induce ⍺1AMPK invalidation specifically in the endothelium. 3 weeks after the last Tamoxifen injection, mice were sacrificed and endothelial cells were immunoprecipitated from lung tissue with dynabeads. ⍺1AMPK gene expression was detected by TaqMan qPCR on both isolated endothelial cells and supernatants. (**a**) Validation of endothelial ⍺1AMPK deletion’s extent in e-AMPK KO. ⍺1AMPK expression detected by TaqMan qPCR in immunoprecipitated endothelial cells. (**b**) Validation of endothelial ⍺1AMPK deletion’s specificity. ⍺1AMPK expression detected by TaqMan qPCR on lysates of lung tissue depleted of endothelial cells. (**c**) Time course of canagliflozin plasma levels in mice treated by oral gavage, 100 mg/kg, during indicated time. The data are mean ± SEM, n = 3 to 5/group. The data were analyzed using one-way ANOVA test.

To better delineate the role played by endothelial α1AMPK in protecting against SCLS, we first generated a mouse model in which endothelial α1AMPK was conditionally and specifically deleted in the endothelium. With this aim in mind, mice expressing the Tamoxifen-responsive conditional Cre-ERT2 fusion protein under the Cdh5 promoter (Cdh5Cre^+/−^) control were crossed with mice expressing LoxP-flanked PRKAA1 gene (α1AMPK^fl/fl^). The Cdh5Cre^−/−^/α1AMPK^fl/fl^ (e-AMPK WT) littermates were employed as controls. To validate the efficacy and specificity of Tamoxifen-induced α1AMPK deletion, α1AMPK gene expression was measured using TaqMan qPCR on endothelial cells that were immunoprecipitated from lung tissues. Results demonstrate 60% α1AMPK depletion in endothelial cells that were isolated from e-AMPK KO, in comparison with e-AMPK WT mice (Fig. 1a). Notably, this invalidation was not observed in immunoprecipitated supernatant that contained non-endothelial cells (Fig. 1b).

### Canagliflozin protects against LPS-induced vascular leakage via endothelial α1AMPK-dependent mechanisms

**Figure 2:**
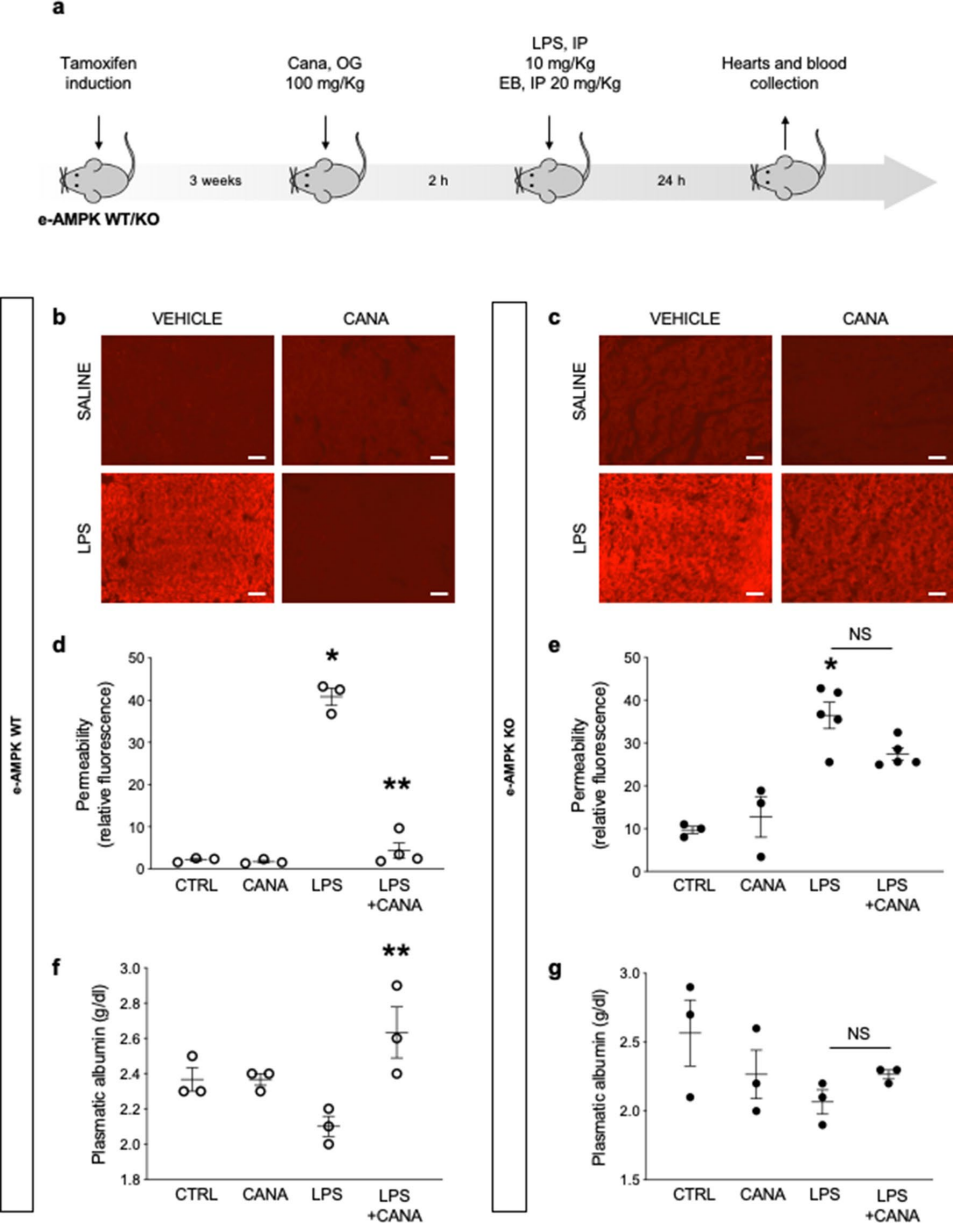
Canagliflozin protects against lipopolysaccharide induced capillary leak syndrome via endothelial ⍺1AMPK dependent mechanisms. (**a**) Schematic representation of the experimental protocol for in vivo permeability assessment. e-AMPK WT/KO mice were intraperitoneally administered Tamoxifen (500 µg/mice) for five consecutive days in order to induce ⍺1AMPK invalidation specifically in the endothelium. 3 weeks after the last Tamoxifen injection, mice were treated with canagliflozin (Cana) (100 mg/Kg) by oral gavage, two hours before being submitted to lipopolysaccharide (LPS) treatment (sublethal doses, 10 mg/kg) by intraperitoneal (IP) injection. Evans Blue Dye (EBD) was administered by IP simultaneously with LPS. (**b**–**e**) Cardiac vascular permeability was assessed via EBD fluorescence quantification on myocardial sections of hearts sampled 24 h after injection of LPS or saline vehicle. Representative images (**b**, **c**) and quantifications (**d**, **e**) are shown. Scale bar, 200 µm. (**f**, **g**) Plasmatic albumin levels were measured on blood samples collected 24 h after injection of LPS or saline vehicle. The data are mean ± SEM, n = 3 to 5/group. *p < 0.05 is relative to control saline group, **p < 0.05 is relative to LPS treated group. NS = nonsignificant. The data underwent two-way ANOVA.

We next investigated whether canagliflozin treatment could prevent LPS-induced vascular leakage in septic mice. Canagliflozin was administered by oral gavage at a dose of 100 mg/Kg, thereby reaching the clinically relevant plasma concentrations of ∼10 µM throughout the 24-h experiment duration, as validated by liquid chromatography-mass spectrometry (Fig. 1c)^33^. Endotoxemia was induced by intraperitoneal injections of sublethal doses of lipopolysaccharide O55:B5 (LPS), an endotoxin produced by Escherichia coli (E. Coli). Our experimental setup is summarized in Fig. 2a. Capillary leak was monitored using Evans Blue Dye (EBD), as previously reported^5^. As expected, EBD detection on myocardial sections of e-AMPK WT mice submitted to LPS was indicative of relevant capillary leakage (Fig. 2b and d). These data were reinforced by measuring plasmatic albumin levels, which clearly revealed that LPS treatment was associated with reduced albuminemia, probably due to albumin leakage (Fig. 2f). Importantly, canagliflozin administration drastically reduced LPS-induced myocardial edema and maintained albumin plasma levels, which is possibly indicative of reinforced vascular barrier function (Fig. 2b, d and f). Finally, similar experiments performed in e-AMPK KO mice demonstrated that canagliflozin protection was abrogated in the absence of endothelial α1AMPK. Indeed, endotoxemia-induced myocardial edema and albumin leakage persisted despite canagliflozin treatment in e-AMPK KO animals (Fig. 2c, e and g). Taken all results together, these data demonstrate that canagliflozin is indeed able to protect septic mice against LPS-induced vascular leakage, based on an endothelial α1AMPK-dependent mechanism.

### Canagliflozin activates α1AMPK/p38 MAPK/HSP27 pathway in HMECs

**Figure 3:**
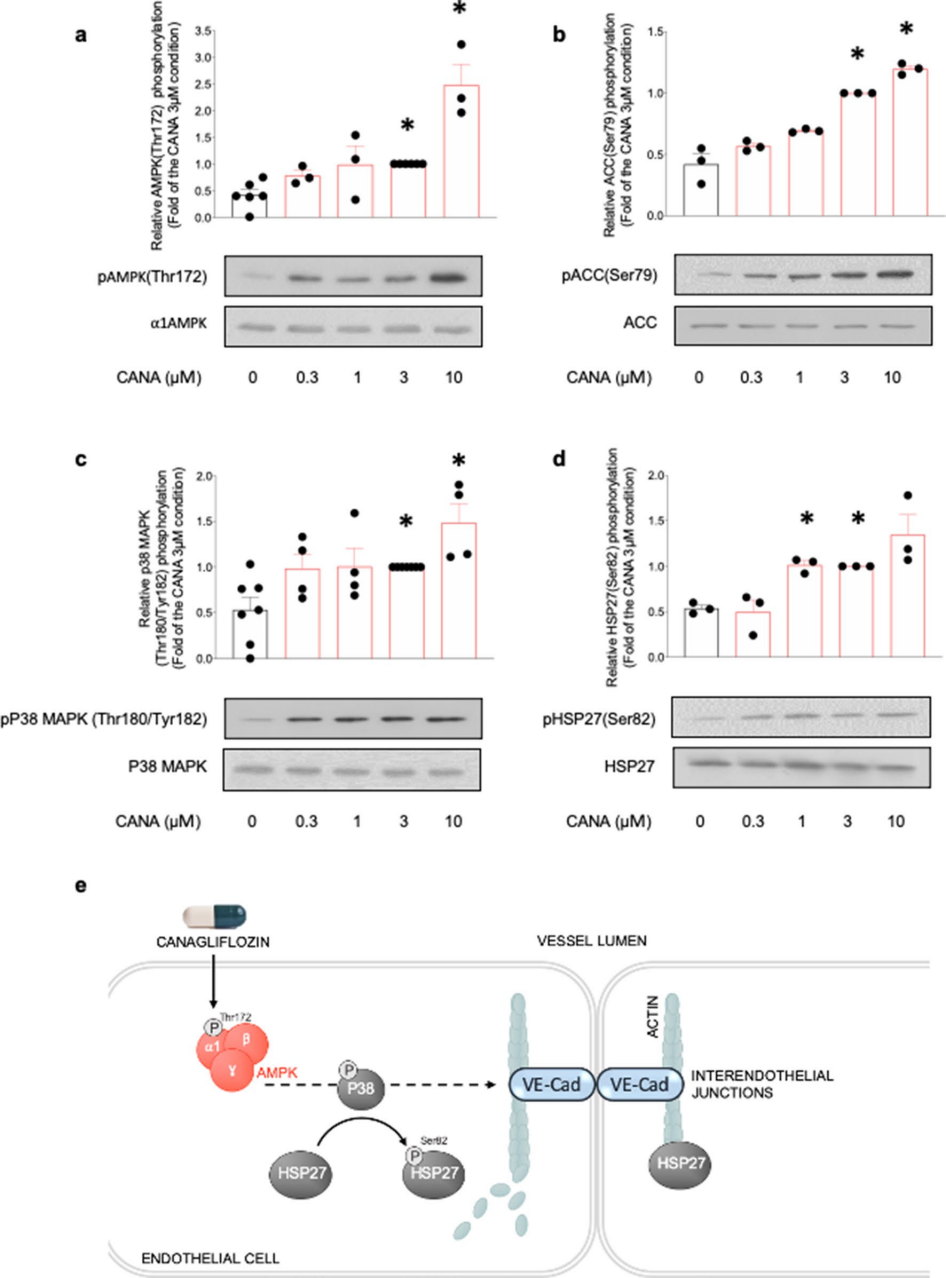
Canagliflozin activates the ⍺1AMPK/p38MAPK/HSP27 pathway in HMECs. HMECs were treated with canagliflozin (Cana) for the indicated concentrations during one hour. Cell lysates were submitted to western blot analysis and probed with total and phosphorylated (**a**) ⍺1AMPK (Thr172), (**b**) ACC (Ser79), (**c**) p38 MAPK (Thr180/Tyr182) and (**d**) HSP27(Ser82) antibodies, (**e**) Molecular mechanisms underlying the protective action of canagliflozin on interendothelial junctions. Representative western blots and quantification are shown. Data are fold of the 3 µM condition and expressed as mean ± SEM (3 to 6 biological replicates for each condition). *p < 0.05 is relative to untreated HMECs. The data underwent one-way ANOVA.

To better understand the molecular mechanisms underlying canagliflozin protection of endothelial barrier function, we assessed the impact of canagliflozin treatment on AMPK activation and its downstream p38MAPK/HSP27 pathway in human endothelial cells. The p38MAPK/HSP27 pathway is known to mediate AMPK-dependent stabilization of inter-endothelial junctions by reorganizing the actin cytoskeleton^15,25,34^. Indeed, HSP27 is an actin-capping protein that inhibits actin polymerization by binding the microfilaments positive ends. HSP27 phosphorylation downstream of the p38MAPK releases HSP27 from actin, thereby enabling further filament polymerization in order to reinforce IEJ anchorage. Since SCLS primarily occurs at the level of capillaries and post-capillary venules, experiments were performed on human endothelial cells derived from the microcirculation (HMECs). Results indicate that canagliflozin dose-dependently activates AMPK, as assessed via phosphorylation of AMPK (Thr172) and its bona fide substrate acetyl-CoA carboxylase (ACC) (Ser79) (Fig. 3a, b). Furthermore, canagliflozin significantly increases phosphorylation of both p38 MAPK (Thr180/Tyr182) and HSP27 (Ser82), and this in a dose-dependent manner (Fig. 3c, d). Supplementary Fig. 1 shows that incubation of HMECs with 3 µM canagliflozin for increasing time periods also resulted in a significant and sustained AMPK activation, as represented by both AMPK (Thr172) and ACC (Ser79) phosphorylation. Taken together, these results demonstrate that clinically relevant canagliflozin concentrations do indeed activate the α1AMPK/p38MAPK/HSP27 pathway in HMECs, thereby potentially reinforcing inter-endothelial junctions by modulating actin cytoskeleton organization (Fig. 3e).

### Canagliflozin-induced AMPK activation protects VE-Cad organization and endothelial barrier function against LPS injury

**Figure 4:**
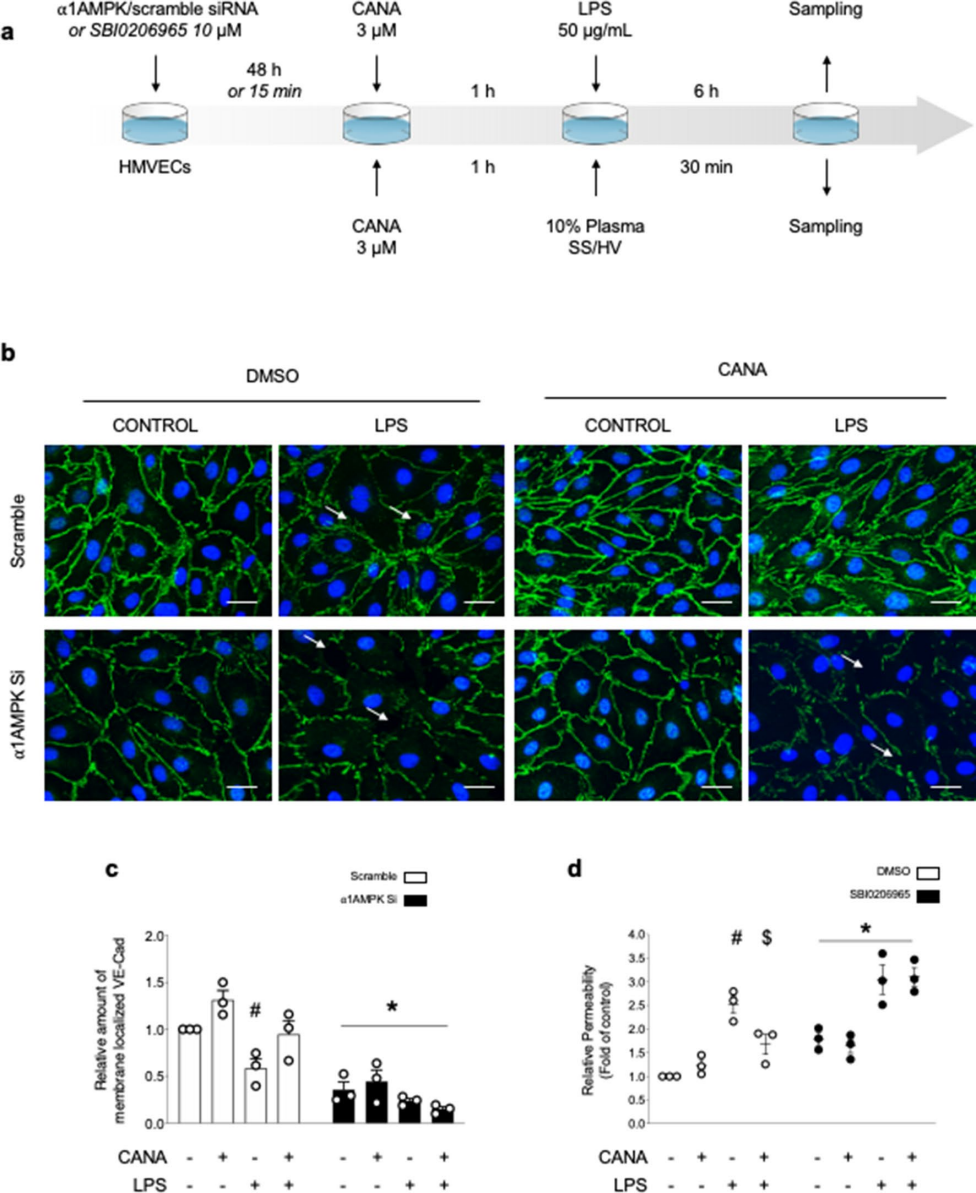
AMPK activation by Canagliflozin protects VE-Cad organization and endothelial barrier function against LPS injury. (**a**) Schematic representation of the experimental protocol. For (**b**, **c**), HMECs were transfected with scramble or ⍺1AMPK targeting siRNA (50 nM) for 48 h, then treated with canagliflozin (Cana) (3 µM) or DMSO for one hour, before adding lipopolysaccharide (LPS) or vehicle (50 µg/mL) for 6 h. For (**d**), SBI compound was incubated for 15 min, then canagliflozin (3 µM) or DMSO were incubated for one hour, before adding LPS (50 µg/mL) for 6 h. (**b**, **c**) VE-Cad immunostainings were performed on HMECs treated according to the protocol detailed in (**a**). Representative images (**b**) and quantifications (**c**) are shown. Intercellular gaps are indicated by white arrows. Nuclei were stained with DAPI. Scale bar, 50 µm. **(d)** Endothelial permeability in response to canagliflozin, LPS challenge, and AMPK inhibitor SBI0206965. HMECs were grown on gelatin-coated Transwell inserts for 72 h and treated according to the protocol detailed in (**a**). Data are expressed as mean ± SEM (3 biological replicates for each condition). ^#^*p* < 0.05 is relative to respective non-treated HMECs, ^$^*p* < 0.05 is relative to LPS-only treated HMECs, and *p < 0.05 is relative to cells treated with DMSO. The data underwent two-way ANOVA.

Next, we investigated the impact of canagliflozin on VE-Cad, *i.e.,* the junctional protein known to be the gatekeeper of endothelial barrier function^12^. Therefore, immunostainings were performed on HMECs, either depleted or not in α1AMPK^25^, before being treated with canagliflozin (3 µM) and LPS (50 µg/mL) (Fig. 4a). Figure 4b illustrates that the continuous peripheral staining of VE-Cad under basal conditions appears to be disorganized in response to LPS treatment. This is associated with the formation of intercellular gaps. In contrast, canagliflozin likely strengthens VE-Cad anchorage within the plasma membrane, preserving its organization and preventing the formation of intercellular gaps in response to LPS. The response to canagliflozin treatment is abrogated in α1AMPK-deficient cells, as confirmed by VE-Cad signal quantifications (Fig. 4b and c). Finally, the impact of canagliflozin-induced AMPK activation on the endothelial barrier function was evaluated in vitro by measuring the clearance of HRP-coupled streptavidin through the HMECs monolayer (Fig. 4d). Because cellular transfection affects by itself the barrier integrity, we employed the pan-AMPK inhibitor SBI0206965 to abrogate AMPK activation. As expected, LPS treatment was revealed to increase endothelial permeability, whereas canagliflozin was demonstrated to protect against LPS-induced endothelial barrier disruption. The SBI0206965 compound, when given alone, was shown to significantly impair endothelial barrier function, whereas this agent completely abolished canagliflozin-induced protection. Of interest, these results are remarkably supported by our previous data demonstrating that α1AMPK is essential in both maintaining expression and architecture of IEJs under basal conditions, and mediating the protective effect of 991 compound, its best pharmacological activator, against IEJs disruption caused by LPS insult^25^.

### Canagliflozin-induced AMPK activation protects VE-Cad integrity in HMECs challenged with human septic plasma

**Figure 5:**
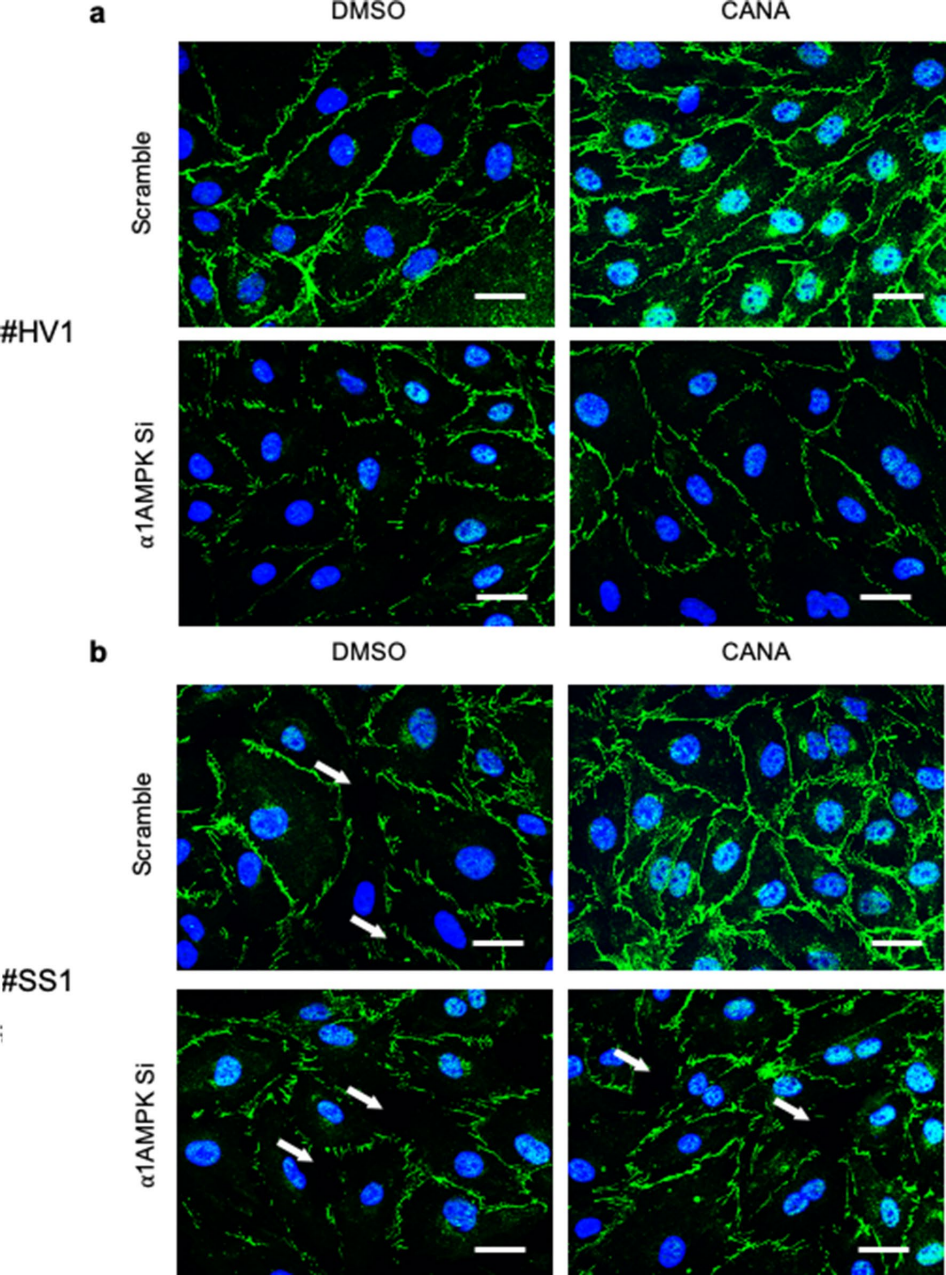
AMPK activation by Canagliflozin protects VE-Cad integrity in HMECs challenged with human septic plasma. VE-Cad immunostainings performed on HMECs. HMECs were transfected with scramble or ⍺1AMPK targeting siRNA (50 nM) for 48 h, before being treated with canagliflozin (3 µM) or DMSO for one hour, then incubated with 10% plasma of (**a**) healthy volunteers (HV) or (**b**) septic shock patients (SS) for 30 min. Typical examples of pictures are shown. The experiment has been repeated with 4 different donors for each group (see Supplemental Fig. 2). Intercellular gaps are indicated by white arrows. Nuclei were stained with DAPI. Scale bar, 50 µm.

Finally, in order to reinforce the translational perspectives of our work, the effects of canagliflozin were evaluated on HMECs incubated with human plasma collected from either control healthy volunteers (HV) or septic shock patients (SS) (Fig. 5a and b). Supplementary Fig. 2 shows experiments performed with supplemental donors. Clinical characteristics of healthy donors and septic shock patients are summarized in Supplementary Tables 1 and 2. Immunostainings show that both HV and SS plasma affect VE-Cad architecture, with SS plasma inducing higher VE-Cad disruption, as represented by discontinuous jagged signals and intercellular gaps formation. Of major interest, canagliflozin importantly preserved VE-Cad integrity and linear organization, while slightly enhancing its membrane expression in HMECs exposed to both HV and SS plasma. On the other hand, α1AMPK depletion was associated with reduced, disrupted VE-Cad signal, and drastic decrease of canagliflozin protective effects. These, however, also seem to involve AMPK-independent mechanisms, since their abrogation appears inconstant in AMPK depleted cells.

## Discussion

Our work highlights canagliflozin’s protective effects on endotoxemia-induced vascular hyperpermeability and demonstrates that endothelial α1AMPK and its downstream p38MAPK/HSP27/VE-Cad regulatory pathway are involved in this protection. During the past decade, canagliflozin, along with other SGLT2i, have emerged as antidiabetic drugs that exhibit remarkable cardiovascular protection^30,31,35–37^, which is not fully explained by their blood glucose-lowering properties^38–40^. Extensive clinical studies are currently conducted to further characterize this protective action. Emerging hypotheses notably suggest that glucosuria and natriuresis, decreased inflammation, or reduced oxidative stress may all contribute to improve cardiovascular function^40^. Interestingly, the diuretic effects of SGLT2i have been shown to selectively reduce interstitial edema with minimal depletion of circulating blood volume^41–43^. Beyond postulating that endothelial barrier integrity possibly plays a significant role in this particular SGLT2i feature, such integrity appears particularly relevant in the SCLS setting.

Here, we have demonstrated that canagliflozin-induced microvascular protection depends, at least to some extent, on endothelial α1AMPK activation. While the SGLT2-induced AMPK activation is attracting growing interest, the hypothesis that this kinase mediates SGLT2’s cardiovascular protective effect is still incompletely explored. Tampering inflammation^44–46^, reducing oxidative stress^47–49^, regulating nitric oxide production^45,50,51^, or preventing energy depletion^45,52,53^ are overlapping cardiovascular protective mechanisms of SGLT2i and AMPK. Our data combined with recent findings supporting an empagliflozin-induced AMPK-dependent protection of microvascular barrier function^53^ enable us to postulate that the endothelial barrier regulation as induced by AMPK activation also represents a key mechanism contributing to the SGLT2i–related cardiovascular protection.

One limitation of our study is that in our model, canagliflozin treatment was administered before LPS challenge. This protocol does, thus, not reflect the clinical reality of sepsis. The canagliflozin impact should further be evaluated and figured as a treatment of declared sepsis. A recent study demonstrating improved survival of septic mice subsequently treated with SGLT2i supports that promising results may reasonably be expected^54^. This perspective, however, raises several issues and questions. It should, first, be determined whether other SGLT2is could exert a similar protective mechanism. Indeed, although canagliflozin was initially described to activate AMPK more robustly compared to other SGLT2i^29^, both empagliflozin and dapagliflozin were subsequently reported to activate AMPK in vivo in both mice total heart samples^45,46^ and cardiac fibroblasts^46^. Moreover, empagliflozin was proven particularly beneficial for microvascular barrier function^53^ and against sepsis injury^45,54^. Therefore, we believe that vascular barrier protection would not be restricted to canagliflozin. Second, SGLT2i administration could be further optimized in order to avoid *per os* formulations for intensive care unit (ICU) settings. In this respect, it is worth mentioning that the feasibility of intravenous canagliflozin administration has been demonstrated recently^50^. Finally, owing to the heterogeneity of clinical sepsis presentations and based on the increasing relevance attached to genetic variants concerning host septic responses, it is unlikely that all septic patients would benefit to the same extent from receiving SGLT2i inhibitors. Dynamic protocols reflecting the integrity of the microcirculation—i.e., orthogonal polarization spectral imaging^55^—could be useful for early identifying patients that are most likely to respond to this new therapeutic approach.

Given the urgent need for therapies targeting SCLS^18^, along with the emerging cardiovascular protective role of SGLT2i, we strongly believe that these aforementioned findings will likely help better link these two research fields and ultimately provide a promising therapeutic approach for SCLS. It must additionally be mentioned that SGLT2i have been recently approved in other indications than diabetes such as heart failure with reduced ejection fraction, extending their clinical applications and daily uses.

## Conclusion

This study highlights endothelial barrier protection by the SGLT2 inhibitor canagliflozin during sepsis, along with α1AMPK/p38MAPK/HSP27/VE-Cad pathway to play a key role in this effect. Canagliflozin could be considered a new therapeutic option in sepsis-induced capillary leak syndrome.

## Methods

### Materials and reagents

Tamoxifen (#T5648), LPS O55:B5 (#L2880), SBI0206965 (#SML1540), TMB substrate (#T040), Evans Blue (#E2129), and Transwell inserts (#3413) were from Sigma-Aldrich (Overijse, Belgium). Collagenase I (#17018029), anti-rat immunoglobulin G–coated magnetic beads (#11035), Cells-to-CT 1-Step TaqMan kit (#A25603), PRKAA1 FAM probe (#Mm01296696_m1), RPL32 VIC probe (Mm02528467_m1), siRNA negative control (#AM4635), siRNA PRKAA1 (#AM51334), and Lipofectamine RNAiMAX reagent (#13778-150) were purchased from Thermo Fisher Scientific (Waltham, MA, USA). We also used Canagliflozin (#HY-10451, Medchem Express, Monmouth Junction, NJ, USA), and HRP-coupled streptavidin (#15:1000, DY998; R&D Systems, Minneapolis, MN, USA). The antibodies employed were rat anti-mouse platelet/endothelial cell adhesion molecule-1 (PECAM-1, #553370; BD Pharmingen) α1AMPK (#MA5-15815; Thermo Fisher Scientific), phospho-AMPK Thr172 (#2535; Cell Signaling Technology, Danvers, MA, USA), phospho-ACC S79 (#3661; Cell Signaling Technology), p38 MAPK (#9212; Cell Signaling Technology), phospho-p38 MAPK T180/Y182 (#9211; Cell Signaling Technology), heat shock protein of 27kDa (HSP-27) (#2402; Cell Signaling Technology), phospho-HSP27 S82 (#44534; Life Technologies, Thermo Fisher Scientific), eEF2 (#PA5-17794; Thermo Fisher Scientific), secondary horseradish peroxidase (HRP)-conjugated antibodies (#A0545; Sigma-Aldrich or #554002; BD Biosciences, San Jose, CA, USA), and Alexa Fluor-coupled secondary antibodies (#A21206; Invitrogen).

### Mice and breeding

All animals were housed with a 12-h/12-h light/dark cycle, with the dark cycle occurring from 6.00 p.m. to 6.00 a.m. Mice were observed daily with free access to water and standard chow. C57BL/6J males (age 8-12wk) were purchased from the Janvier labs (Le Genest Saint Isle, France). C57BL/6J Cdh5-iCreERT2 mice^56^ were kindly provided by Ralf Adams, and crossed with mice carrying a floxed allele of PRKAA1 gene (PRKAA1^fl^/^fl^, #014141, the Jackson Laboratory). Cdh5-iCreERT2+ /− PRKAA1^fl^/^fl^ mice were administered Tamoxifen (500 μg, intraperitoneally) for five consecutive days at 8 weeks, and used for experiment three weeks after the last Tamoxifen injection. The animals were maintained under a 12:12-h light–dark cycle with free access to food and water.

### Lung endothelial cell isolation and model validation

Mouse lungs were harvested, rinsed and incubated in Dulbecco's modified Eagle's medium containing 2 mg/mL collagenase I for 45 min at 37 °C. The cells were then centrifuged at 1000 g for 5 min at 4 °C, resuspended in buffer 1 (0.1% bovine serum albumin, 2 mM EDTA, in PBS), and incubated with anti-rat immunoglobulin G–coated magnetic beads precoupled with rat anti-mouse PECAM-1 antibody for 30 min at 4 °C in an overhead shaker. Beads were separated from the solution with a magnetic particle concentrator (Dynal MPC-S). The supernatant was kept and the beads were washed five times with buffer 1. Cells-to-CT 1-Step TaqMan kit was used for both the supernatant and the purified endothelial cells, before performing Taqman PCR technology for α1AMPK expression quantification. Data were analyzed with the 2 (-Delta Delta C(T)) method^57^, and expressed as fold of controls.

### In vivo model of endotoxemia, cardiac permeability assessment, and plasmatic measurements

Canagliflozin was suspended in saline solution containing 0.5% carboxymethylcellulose and 0,025% Tween-20 and administered by oral gavage (100 mg/Kg, 10µL/g), as described previously^29^. Endotoxemia was induced by intraperitoneal (IP) injections of either LPS (10 mg/Kg) or saline vehicle. For myocardial permeability studies, Evans Blue Dye (EBD) was administered by IP injections (20 mg/Kg) and used to quantify albumin extravasation, as described previously^5^. For heart sampling, animals were euthanized with IP injections of pentobarbital (300 mg/Kg) following 24 h. Vascular leakage, corresponding to the dye amount within the extravascular compartment, was quantified using image J software (Wayne Rasband, National Institutes of Health, Bethesda, MD), as the relative fluorescence (594 nm) surface on frozen Sects. (6-um thick). For blood collection, mice were bled under ketamine and xylazine anesthesia from the retro-orbital plexus. Plasma was obtained by centrifugation at 3000 g for 15 min, followed by 14800 g for 3 min. Albumin was measured by colorimetric method, using FUJI Dry-Chem NX500 biochemical system. For plasma quantification, canagliflozin was analyzed by HPLC–MS/MS system consisting in a Xevo TQ-S mass spectrometer (Waters) coupled to an Acquity UPLC Class H system (Waters). Dapagliflozin was employed as internal standard. The chromatographic separation was performed using a Kinetex C18 HPLC column. Multiple reaction monitoring analysis was performed following electrospray ionization in positive mode.

### Human plasma sampling

Patients with septic shock admitted at *Cliniques universitaires Saint-Luc*, Brussels, were included in the analysis. Septic shock was defined as a sepsis with vasopressor therapy needed to elevate mean arterial blood pressure (MAP) ≥ 65 mmHg, and lactate > 2 mmol/L, despite adequate fluid resuscitation of 30 mL/kg of intravenous crystalloid within 6 h. Patients on therapeutic oral or parenteral anticoagulation therapy (including heparins, fondaparinux, vitamin K antagonist, or novel oral anticoagulants), with previous history of thrombocytopenia (< 100,000 platelets/mm3), recent (less than 1 month) chemotherapy, cirrhosis (Child Pugh > A), or recent (less than 48 h) major surgery, and those patients with active inflammatory disease, hemophilia, or other coagulopathy were excluded from the analysis. The control group comprised healthy volunteers. For the experimental group, blood samples were obtained in the ICU using the routinely inserted central venous catheter, within 48 h of septic shock diagnosis. For the control group, blood samples were collected by venous puncture. Platelet-rich-plasma (PRP) was obtained after centrifugation at 800* g* for 5 s, followed by centrifugation at 100* g* for 5 min. Next, platelets were pelleted by centrifugation at 400 g for 10 min. Apyrase and Integrilin were added to limit platelet activation during the preparation.

### Cell culture and treatments

Human Microvascular Endothelial Cells (HMECs) were purchased from Lonza (#cc-2543) and cultured according to the manufacturer’s recommendations, using EGM-2 MV microvascular endothelial cell growth medium (#cc-3202, Lonza, Verviers, Belgium) containing 1% penicillin–streptomycin, at 37 °C and 5% CO_2_ in a humidified incubator. The cells were subcultured when reaching 80% confluence and used until subculture number 7. Medium deprivation was performed with minimal medium for two hours prior to treatment or experimentation.

### siRNA transfection

For endothelial α1AMPK silencing, HMECs were seeded the day before transfection to reach 60% confluence within 24 h. Reverse transfection was then performed for 48 h with a control non-targeting siRNA construct (50 nM) or a siRNA specifically targeting PRKAA1 (50 nM). This was performed using a lipofectamine RNAimax transfecting reagent, which adhered to the manufacturer’s instructions.

### Western blotting

Protein content was measured by means of the Bradford method, using bovine serum albumin (BSA) as reference. Proteins (15 μg) were separated by sodium dodecyl sulfate–polyacrylamide gel electrophoresis, then electroblotted. Membranes were probed with the primary antibody overnight at 4 °C in adequate dilution; α1AMPK (1:1000), phospho-AMPK Thr172, (1:1000), phospho-ACC (1:5000), p38MAPK (1:1000), phospho-p38 MAPK T180/Y182 (1:1000), HSP27 (1:1000), phospho-HSP27 S82 (1:1000), and eEF2 (1:1000). Bound antibodies were detected by means of chemiluminescence. Loading was controlled with anti-eukaryotic elongation factor 2. Quantification was assessed using Image J software. Each experiment was repeated at least three times. The original and unprocessed gels/images have been included in Supplementary Figs. 3 and 4.

### In vitro transwell assay

For the endothelial permeability assay, HMECs (10^5^cells/well) were seeded on gelatin-coated Transwell inserts of 24-well plates, in 250μL complete with Endothelial Cells Growth Medium MV. They were then incubated for 72 h at 37 °C and with 5% CO2. The cells were incubated in free M200 medium for two hours before stimulation. The cells were then incubated with the different compounds, as indicated in the figure legends. After treatment, the upper chamber medium was replaced by 300μL of M200, containing HRP-coupled streptavidin. The lower chamber medium was collected after 10-min incubation at 37 °C, and every condition was aliquoted in triplicate. The TMB substrate was added for 10 min, and 2 N H_2_SO_4_ was applied to stop the reaction before acquiring 450 nm absorption in an Elisa reader. Resultant absorption intensity values were normalized over the vehicle control condition. Each experiment was repeated three times.

### In vitro immunofluorescence staining and image analysis

HMECs were seeded on non-coated glass coverslips at a density of 20 × 10^3^cells/cm^2^, 72 h before treatment. After treatment, cells were fixed in 4% paraformaldehyde, permeabilized with 0.3% triton X-100 for 10 min, and then blocked with 10% BSA for 45 min. Cells were then stained as previously described^5^, using VE-Cad primary antibodies (1:25) and Alexa Fluor-coupled secondary antibodies (1:1000). Nuclei were stained using 4’,6-diamidino-2-phenylindole (DAPI). Stainings were visualized under a Zeiss Imager Z1 microscope that was equipped with an ApoTome device. Pictures were acquired using an ×20 objective. Each experiment was repeated three times.

Quantitative image analysis was performed on uncompressed images (native format: zvi) with Fiji 1.52n on MacOS (10.14.5). One image was analyzed per condition and for each experiment. Intercellular junctions, evidenced by VE-Cad staining, were automatically delimited using a fixed-value threshold method. The stained area was quantified, and the mean signal intensity was calculated with this section. Stained membrane segments were subsequently detected using the Analyze Particles ad Skeletonize tools, and automatically counted. For normalization purposes, all images’ nuclei were automatically counted using a threshold method and the analyze particles tool.

### Ethics approval and consent to participate

The study was approved in 2018 by the Ethical Review Board of Cliniques universitaires Saint-Luc/UCLouvain (V1 04/12/2018). All methods were carried out in accordance with relevant guidelines and regulations. All participants provided written informed consent.

Animal handling and experimental procedures were approved by local authorities at UCLouvain (Comité d’éthique facultaire pour l’expérimentation animale, 2016/UCL/MD/027) and performed in accordance with the Guide for the Care and Use of Laboratory Animals, published by the US National Institutes of Health (NIH Publication, revised 2011). All the authors complied with the ARRIVE guidelines.

### Statistical analyses

The sample size was not pre-determined based on statistical analysis, and it was chosen according to previous publications. Statistical analyses were conducted using SPSS v.25 Software (IBM Corp., Armonk, NY, USA), and graphs were build using GraphPad Prism 7.0 (GraphPad Software, La Jolla, CA, USA). All tests were two-sided, with statistical significance set at the 0.05 probability level. Data were expressed as mean ± standard deviation. Means were compared using unpaired Student’s *t*-test or a one-way or two-way analysis of variance, as appropriate. The Bonferroni correction was applied for multiple comparisons.

## Supplementary Information

Supplementary Information.

## Data Availability

The datasets used during the current study are available from the corresponding author on reasonable request.

## Abbreviations

ACC: Acetyl-CoA carboxylase
AJs: Adherens junctions
AMPK: AMP-activated protein kinase
eEF2: Eukaryotic elongation factor 2
HMEC: Human dermal microvascular endothelial cell
HSP27: Heat shock protein 27
IEJs: Inter-endothelial junctions
LPS: Lipopolysaccharide
p38 MAPK: P38 mitogen-activated protein kinase
SCLS: Sepsis capillary leak syndrome
SGLT2i: Sodium-glucose co-transporter 2 inhibitor
siRNA: Small interfering RNA
VE-Cad: VE-cadherin
