# Supplementary material for: Canagliflozin protects against sepsis capillary leak syndrome by activating endothelial α1AMPK
Source: Sci Rep. 2021 Jul 1;11:13700. doi: 10.1038/s41598-021-93156-1 (PMC8249425; doi:10.1038/s41598-021-93156-1)
Supplement: Supplementary file 1 — Supplementary Information. [file 41598_2021_93156_MOESM1_ESM.pdf]

Supplementary Table 1

Donor	HV1	HV2	HV3	HV4
Age	31	39	25	24
Sex	female	female	female	female
Weight (kg)	62	57	58	59
Height (cm)	169	170	162	156
Medication	No	No	No	No

Supplementary Table 1. Clinical characteristics of healthy donors. Related to Fig. 5a.

Supplementary Table 2

Patient	SS1	SS2	SS3	SS4
ICU admission	12/12/19	1/02/20	9/02/20	16/07/20
Age	58	58	75	70
Sex	female	male	female	female
Weight (kg)	65	105	55	96
Height (cm)	165	180	162	158
Diagnostic<br>Germs	Peritonitis<br>Escherichia coli<br>Clostridium tertium	Erysipelas<br>Streptococcus pyogenes	Peritonitis/Empyema<br>Pseudomonas aeruginosa	Peritonitis<br>Enterococcus faecalis<br>Enterobacter cloacae
Smoking	Yes	No	Yes	No
Arterial hypertension	No	Yes	Yes	No
Diabetes	No	No	No	No
Chronic obstructive pulmonary disease	No	No	Yes	No
Chronic renal failure	No	No	Yes	No
Cancer	No	No	No	No
Anticoagulation	No	Yes	Yes	No
Noradrenaline dosis ug/kg/min	0.36	0.31	0.54	0.9
Dialysis	No	No	No	No
CRP (mg/L)	251.9	346.5	334	67.2
White blood cells (/mL)	33 870	8 970	18 190	14 500
Platelets (/mL)	223	32	105	65
INR (International Normalized Ratio)	1.41	0.96	1.19	2.10
Apache Score	12	17	18	24
SOFA Score	5	15	6	14
Lenght of ICU stay	18	7	6	6
Duration of ventillation (days)	18	0	6	1
Death at 30 days	Yes	No	Yes	Yes

Supplementary Table 2. Clinical characteristics of septic shock patients. Related to Fig. 5b.

Supplementary Figure 1

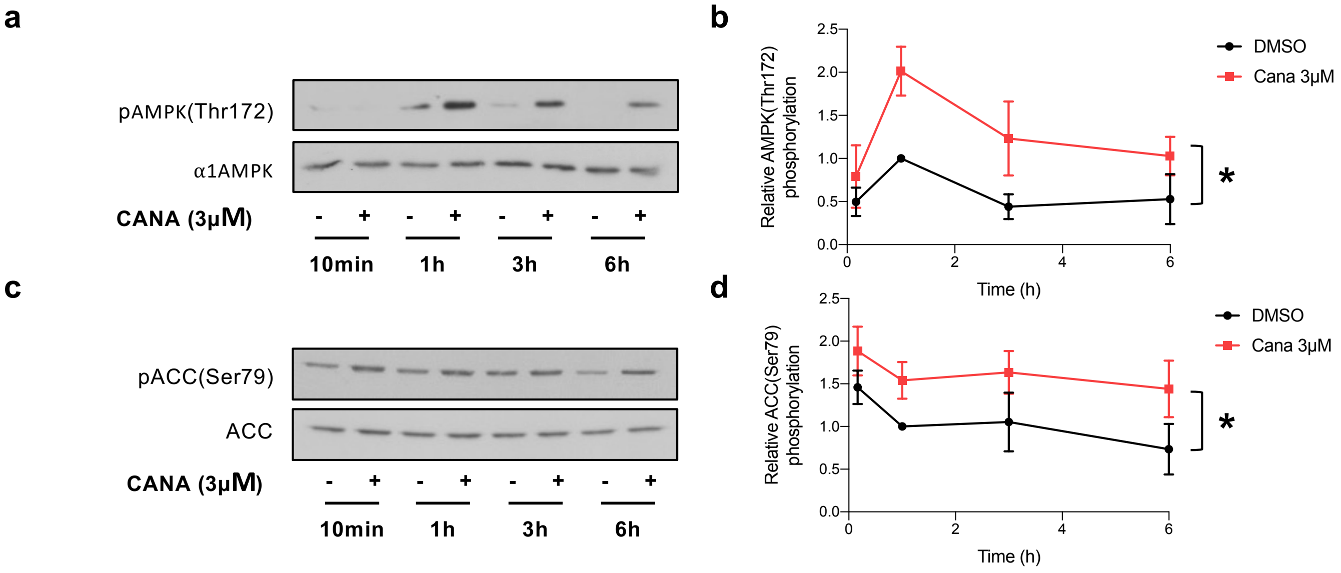

Supplementary Figure 1. HMECs were treated with 3 μM canagliflozin (CANA) for the indicated times. Cell lysates were submitted to western blot analysis and probed with (a) total and phosphorylated α1AMPK (Thr172) and (b) total and phosphorylated ACC (Ser79) antibodies. Related to Fig. 3a and 3b.

Supplementary Figure 2

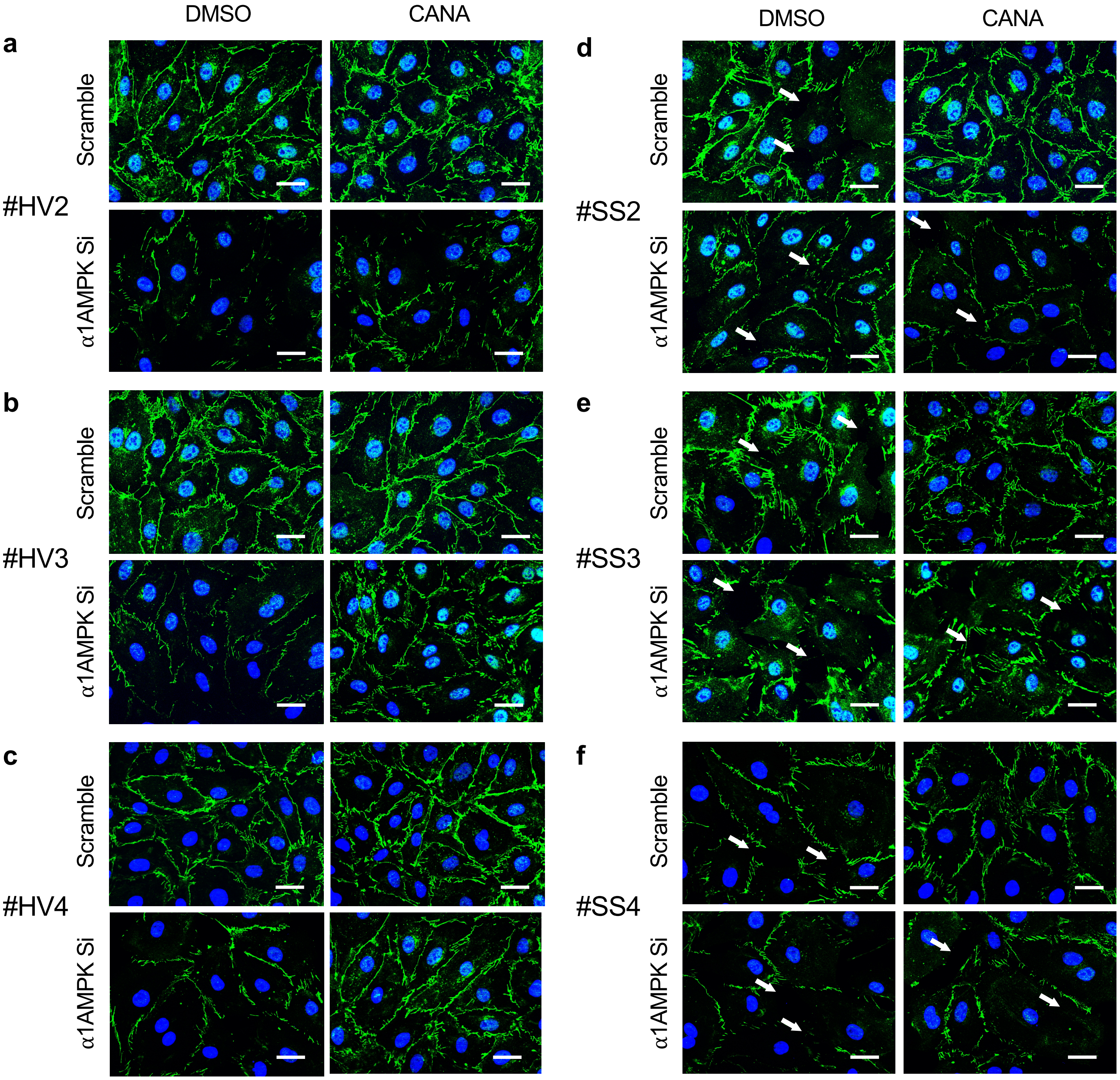

Supplementary Figure 2. AMPK activation by canagliflozin (CANA) protects VE-Cad integrity in HMECs challenged with human septic plasma. VE-Cad immunostainings performed on HMECs. HMECs were transfected with scramble or α1AMPK targeting siRNA (50 nM) for 48 hours, before being treated with CANA 3 µM or DMSO for 1 hour, then incubated with 10% plasma of (a) healthy volunteers (HV) or (b) septic shock patients (SS) for 30 minutes. Intercellular gaps are indicated by white arrows. Nuclei were stained with DAPI. Scale bar, 50µm. Related to Fig. 5.

Supplementary Figure 3

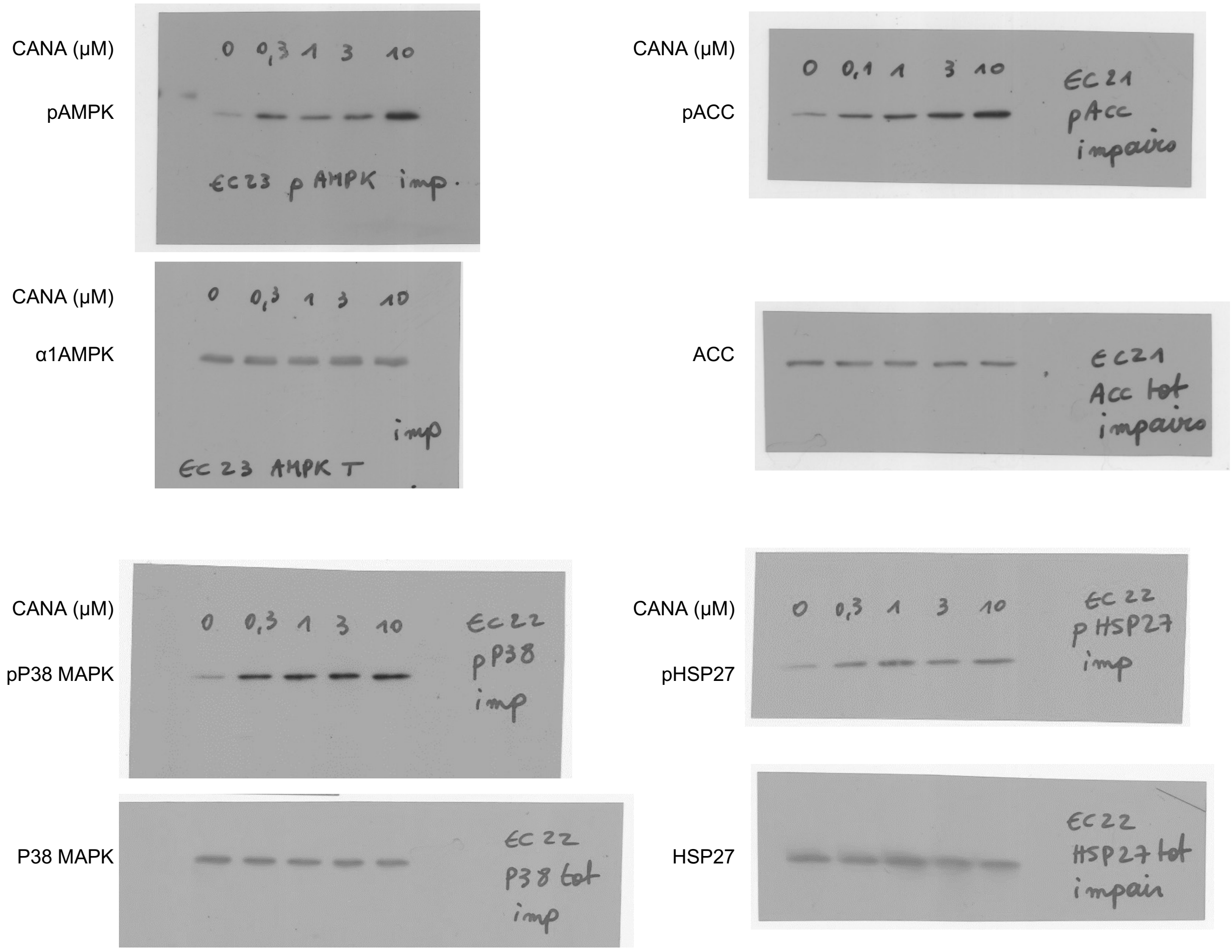

Supplementary Figure 3. The original and unprocessed gels/images illustrated in Fig. 3.

Supplementary Figure 4

CANA (3µM)

pAMPK

α1AMPK

pACC

ACC

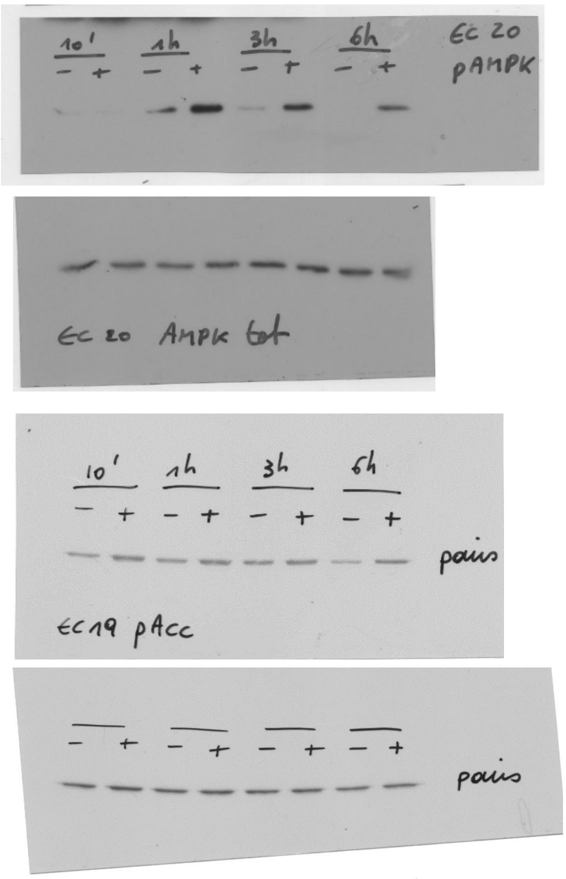

Supplementary Figure 4. The original and unprocessed gels/images illustrated in in Supplementary Fig. 1 .
